# Anti-Inflammatory and Skin Barrier Repair Effects of Topical Application of Some Plant Oils

**DOI:** 10.3390/ijms19010070

**Published:** 2017-12-27

**Authors:** Tzu-Kai Lin, Lily Zhong, Juan Luis Santiago

**Affiliations:** 1 Department of Dermatology, Kaohsiung Chang Gung Memorial Hospital and Chang Gung University College of Medicine, Kaohsiung 83301, Taiwan; tklintklin@gmail.com; 2 California State University, Los Angeles, School of Nursing, 5151 State University Dr, Los Angeles, CA 90032, USA; 3 Dermatology Service & Translational Research Unit (UIT), Hospital General Universitario de Ciudad Real, 13005 Ciudad Real, Spain

**Keywords:** plant oil, barrier function, barrier repair, wound healing, inflammation, antioxidant activity, skin aging

## Abstract

Plant oils have been utilized for a variety of purposes throughout history, with their integration into foods, cosmetics, and pharmaceutical products. They are now being increasingly recognized for their effects on both skin diseases and the restoration of cutaneous homeostasis. This article briefly reviews the available data on biological influences of topical skin applications of some plant oils (olive oil, olive pomace oil, sunflower seed oil, coconut oil, safflower seed oil, argan oil, soybean oil, peanut oil, sesame oil, avocado oil, borage oil, jojoba oil, oat oil, pomegranate seed oil, almond oil, bitter apricot oil, rose hip oil, German chamomile oil, and shea butter). Thus, it focuses on the therapeutic benefits of these plant oils according to their anti-inflammatory and antioxidant effects on the skin, promotion of wound healing and repair of skin barrier.

## 1. Introduction

Skin, the largest organ of the body, functions as the necessary interface between the internal and the external environment. Thus, it continuously protects the body from noxious stimuli, e.g., microorganisms, ultraviolet (UV) irradiation, allergens, and irritants. Its unique role and function is a direct result of its structure and makeup, particularly of the most superficial part, the epidermis. The main cellular component of the epidermis includes keratinocytes, but there are also melanocytes, Merkel cells, gamma delta T-lymphocytes, and Langerhans cells. Keratinocytes in the basal layer of the epidermis preserve their ability to proliferate upward to form the spinous layer and the granular layer. Beyond the granular layer, the keratinocytes terminally differentiate into corneocytes in the horny layer. In the outmost part of epidermis, corneocytes (compact keratinocytes without nuclei), together with the intercellular lamellar compartment (lipids), contribute to the structure and function of the stratum corneum (SC).

A PubMed literature search was performed using the following terms: plant oils and atopic dermatitis (AD), skin aging, skin barrier function, skin cancer, and wound healing (WH). Focusing on potential benefits of topically applied plant oils, we chose those that have been previously investigated in human skin, animal skin (mainly murine models of skin disease), or in vitro studies with keratinocytes. The search included clinical use of topical oil plants, but excluded more specific studies related to the biochemical extraction, purification, and modification of these plant oils and their byproducts.

### 1.1. Stratum Corneum Structure and Function

The structure of SC is like a brick wall, in which the corneocytes or “bricks” are surrounded by the intercellular lipid lamellae that act like the “mortar” to maintain both SC integrity and skin permeability barrier [1]. The skin’s barrier function depends mainly on the integrity of the SC. During differentiation, the plasma membrane of outer keratinocytes is replaced by the specialized cornified envelope (CE) of corneocytes. The CE gives corneocytes their rigidity. The development of the CE is attributed to the crosslinking of insoluble proteins (involucrin and loricrin) by transglutaminases. Some of the lipids (precursors of ceramides, free fatty acids (FFAs), and part of cholesterol) are synthesized in the keratinocytes at the stratum granulosum (SG) and then released from the lamellar bodies (LBs) into the SG-SC interface, whereas the remaining lipids are secreted onto the skin surface from the sebaceous glands (sebum). The permeability barrier is provided by the intercellular lipid-enriched matrix, which is composed of ceramides, FFAs, and cholesterol. Following the secretion of LBs, intercellular lipids are enzymatically modified to become the highly hydrophobic and organized lamellar structure. SC lamellar membranes are mostly composed of saturated FFAs of significantly longer chain length, which varies between C16 and C26. The main FFAs in the lamellar membranes are palmitic acid (C16:0) by 10% (mass/mass), stearic acid (C18:0) by 10% (mass/mass), behenic acid (C22:0) by 15% (mass/mass), lignoceric acid (C24:0) by 25% (mass/mass), and hexacosanoic acid (C26:0) by 10% (mass/mass) constitution of the total FFAs in SC [2]. Other FFAs that present less in the SC include oleic acid (C18:1, n-9), eicosapentaenoic acid (C20:5, n-3), arachidonic acid (C20:4, n-6), docosahexaenoic acid (C22:6, n-3), linoleic acid (C18:2, n-6) as well as its derivatives that are linolenic acids [α-linolenic acid (C18:3, n-3), γ-linolenic acid (C18:3, n-6) and dihomo-γ-linolenic acid (C20:3, n-6)] [3]. The C22 and C24 saturated FFAs are present in relatively large amounts among the saturated FFAs, whereas the C18 unsaturated FFAs are the major constituents in unsaturated FFAs. In fact, linoleic acid is the most abundant polyunsaturated fatty acid [4]. Aside from linoleic acid and arachidonic acid, the remaining FFAs can be synthesized in the keratinocytes [5,6,7].

The SC acts as a permeability barrier and an antimicrobial barrier. This antimicrobial barrier is attributed to the weak acidity of skin surface pH, free sphingoid bases generated from epidermal ceramides [8], and antimicrobial peptides within the intercellular compartment. Hydration of the SC is also crucial for the SC integrity and the maintenance of the skin barrier homeostasis. Natural moisturizing factor (NMF) components within the corneocytes contribute to the hydration of the SC. The composition of NMF includes free amino acids, pyrrolidone carboxylic acid, lactic acid, urocanic acid, organic acids, peptides, sugars, urea, citrate, glycerol, etc. Filaggrin, one of the terminal differentiation markers of the epidermis, also aids in SC hydration. Filaggrin is degraded into free amino acids in the SC. These amino acids are further metabolized into hygroscopic derivatives such as pyrrolidone carboxylic acid from glutamine and urocanic acid from histidine. This makes filaggrin one of the major factors influencing the hydration status of the SC.

### 1.2. Atopic Dermatitis and Barrier Function

In the presence of dermatitis, the hydration of the SC decreases and transepidermal water loss (TEWL) increases [9]. In clinical practice, the measure of TEWL is an important indicator of skin barrier function. Additionally, skin dryness (with or without clinical desquamation) is often associated to inferior barrier function [10]. It has been shown that emollient use for eczematous dermatitis such as AD improves barrier function by restoring hydration at SC and reducing TEWL [11]. AD is a common chronic skin inflammatory disease. The pathogenesis of AD is attributed to both epidermal barrier dysfunction and chronic Th2 inflammation within the skin. Impairments in skin barrier function are inevitably present everywhere on skin surface in all AD patients independent of the clinical appearance of the skin. As a result, this impairment of the skin barrier is considered a primary event in AD pathogenesis [12]. Perturbed barrier function largely contributes to the allergic sensitization to both protein antigens and staphylococcal superantigens. Moreover, the inflammation underneath the barrier can alter the differentiation of epidermis, leading to disrupted barrier function. Research has shown that Th2-related cytokines (IL-4) exacerbate skin barrier impairment by modifying the keratinocyte differentiation and lipid synthesis of the intercellular compartment of the SC [13,14]. Therefore, it has been proposed that early interventions to repair the epidermal barrier with the use of appropriate soaps, emollients, or moisturizers may be useful in the control of this chronic disease as well as the prevention of its progression, also known as atopic march [12].

### 1.3. Wound Healing

Wound healing (WH) is a dynamic and tightly regulated process of cellular, humoral, and molecular mechanisms. The process is depicted in four phases: hemostasis, inflammation, proliferation, and tissue remodeling [15]. In the hemostasis phase, the clotting cascade is instantly activated following an injury, creating a temporary wound matrix [16]. The inflammation phase consists of an innate immune response crucial in the breakdown and cleanup of tissue and pathogen debris at the site of injury. Polymorphonuclear neutrophils (PMNs) release reactive oxygen species (ROS) and nitric oxide, facilitate degradation of foreign organisms, and initiate phagocytosis of pathogens. Additionally, PMNs secrete high levels of PMN collagenase, elastase, and matrix metalloproteinases (MMPs), which break down damaged cells and extracellular matrix [17]. Macrophages work through phagocytosis of pathogens and cell debris [18]. Increased numbers of macrophages along with persistent inflammation are observed in chronic wounds [18]. In contrast to acute wounds, where inflammation is crucial in the initial phases of wound repair, chronic non-healing wounds could result from the aberrant inflammatory response in proportion to its intensity and duration [15,16,17,18]. Therefore, inflammation can positively or negatively affect the WH process. Excessive inflammation and/or duration is correlated with increased number of macrophages, resulting in compromised WH outcomes. Additionally, excessive levels of MMPs that are released from PMNs and macrophages, lead to extensive damage of extracellular matrix. This interferes with the normal formation of the scaffold for new cells to migrate and proliferate in wounded areas [19]. Studies of impaired WH models of obese (ob/ob) and diabetic (db/db) mice have shown that the number of macrophages is elevated in those models [20]. Wound closure in obese mice (ob/ob) can be improved by systemic anti-tumor necrosis factor-alpha (TNF-α) treatment through inactivation of macrophages [20]. Similarly, ROS and their oxidative reaction products present in the wound may also play a major role in tissue damage [15,16,17,18]. Although ROS are part of normal regulatory circuits of skin barrier function, inflammation, and WH under physiological conditions, an excess in ROS is detrimental to the WH process [18].

### 1.4. Skin Inflammation and Proliferation

The skin encounters daily onslaught by exogenous stimuli. Noxious stimuli sometimes result in injuries and/or infections, leading to wound, inflammatory dermatoses, skin aging, or skin carcinogenesis. Inflammation takes place in response to these damages to the normal skin barrier. At the molecular level, the inflammatory response participates in a series of complex repair pathways related to the innate immune response, cutaneous differentiation, and skin barrier repair [15]. Initially, upon inflammatory response, the keratinocytes and the innate immune cells such as leukocytes (PMNs, macrophages, and lymphocytes), mast cells, and dendritic cells are activated [15]. Secreted cytokines such as IL-1α, TNF-α and IL-6 induce the chemokines of chemotaxis that attract the immune cells to the site of injury and infection. ROS are produced by activated keratinocytes and immune cells. Immune cells also secrete elastases and proteinases [15]. The inflammatory microenvironment contributes to tissue repair and infection prevention/control. However, the chemokines produced by activated keratinocytes and immune cells are also able to damage the skin tissue in proximity to the target of the inflammatory response. Therefore, the intensity of inflammation and the time to resolution are critical in avoiding or at least limiting damage to normal skin tissue [15]. Thus, modulation of inflammation is important in maintaining skin homeostasis. If the initial acute response fails to resolve the causative factor, then the inflammatory response will continue and the subsequent inflammatory microenvironment will disrupt skin homeostasis. If the dysregulation of inflammatory skin response persists, chronic inflammatory dermatoses such as AD or psoriasis will arise [15,21].

In the epidermis, the metabolism of polyunsaturated fatty acids (PUFAs) is highly active. Linoleic acid, the major 18-carbon n-6 PUFA in normal epidermis, in the epidermis is metabolized via the 15-lipoxygenase pathway mainly into 13-hydroxyoctadecadienoic acid, which possesses anti-proliferative properties [3]. Dietary deficiency of linoleic acid results in a scaly and pruritic skin disorder similar to AD in hairless mice [22]. Arachidonic acid, the second major PUFA in the skin, is another substrate of 15-lipoxygenase, by which it is transformed to 15-hydroxyeicosatetraenoic acid (15-HETE). 15-HETE specifically inhibits leukotriene B4-induced chemotaxis of human PMNs [23]. However, arachidonic acid is mainly metabolized via the cyclooxygenase (COX) pathway into the prostaglandins E(2), F(2α), and D(2) [3]. At low concentrations, the prostaglandins function to modulate skin homeostasis, whereas, at high concentrations, they induce skin inflammation and hyperproliferation of keratinocytes [24]. Moreover, squamous cell carcinoma of skin is the neoplasm that consistently overexpresses COX-2 in the parenchyma and the mesenchyma of premalignant and malignant lesions [25]. Increased levels of prostaglandins E(2) and F(2α) in premalignant and/or malignant epithelial skin cancers are due to the constitutive upregulation of enzymes such as COX-2, causing increased prostaglandin biosynthesis and the downregulation of 15-hydroxy-prostaglandin dehydrogenase (15-PGDH), which is involved in the inactivation of prostaglandins [26]. Thus, topical supplementation with plant oils that provide local cutaneous anti-inflammatory and anti-proliferative metabolites could serve as the monotherapy or as adjuncts to standard therapeutic regimens for the management and prevention of both inflammatory skin disorders and actinic keratoses.

### 1.5. Reactive Oxidative Stress, Skin Aging and Skin Cancer

The aging of our skin can be discussed as two entities: chronological and environmentally- influenced aging [27]. Clinically, chronological and environmentally-influenced aging show skin changes including thinning, loss of elasticity, roughness, wrinkling, increased dryness, and impairment of the skin barrier. Chronological aging depends on a decrease in cellular replacement (senescence) of the epidermis, dermis, and hypodermis, but also from impairment in the remodeling of the extracellular matrix (e.g., collagen bundles and elastic fibers) [28]. The second type of skin aging is mediated by extrinsic factors such as UV radiation, air pollution, smoking, changes in external temperature, and other agents of skin aging exposome [29]. Photoaging by chronic exposure to UV radiation is the best characterized. Clinical signs of photoaging include dyspigmentation (mostly lentigo and freckling), solar elastosis, actinic keratosis, and seborrheic keratosis [30]. Photoaging is attributed to photo-oxidative damage to skin, mainly by high levels of ROS induced by UV radiation [31]. ROS result in collagen degradation and its accumulation in the dermis, also known as solar elastosis. ROS levels are regulated by anti-oxidant enzymes in skin such as superoxide dismutase (SOD), catalase (CAT), and glutathione (GSH). If anti-oxidant defenses are overwhelmed after extensive UV light exposure, ROS production exceeds the capacity of antioxidant defenses in the skin [32]. This causes oxidative stress, which damages skin cells and alters their gene expression, leading to photoaging, but also promoting cutaneous carcinogenesis (non-melanoma and melanoma skin cancers) [33,34,35].

## 2. The Constituents of Plant Oils

Plant oils have long been used on the skin for cosmetic and medical purposes because they have been found to have many positive physiological benefits. For example, plant oil application may act as a protective barrier to the skin by an occlusive effect, allowing the skin to retain moisture, resulting in decreased TEWL values. Additionally, topical products have the benefit of higher bioavailability in the skin and having a localized effect rather than systemic effects. Previous research on plant oils have demonstrated that almond, jojoba, soybean, and avocado oils, when applied topically, mostly remain at the surface of skin, without deep penetration into the first upper layers of the SC [11]. Although triglycerides do not penetrate deeper in SC, glycerol contributes to the SC hydration. Free fatty acids (FFAs), specifically monounsaturated FFAs such as oleic acid, may disrupt skin barrier and act as permeability enhancers for other compounds present in plant oils [36]. Other components such as phenolic compounds and tocopherols exhibit an antioxidant effect and may modulate physiological processes such as skin barrier homeostasis, inflammation, and WH [37,38,39]. When topically applied to hairless mice, sodium dl-α-tocopheryl-6-*O*-phosphate, a water-soluble derivative of vitamin E (dl-α-tocopherol), enhances ceramide synthesis and gene expression of differentiation markers (transglutaminase 1, cytokeratin 10, involucrin, and loricrin) [40]. Phospholipids, another component of plant oils, mainly fuse with the outer lipid layer of the SC, potentially acting as chemical permeability enhancers [41]. In a study of the murine AD model with given dietary phospholipid supplementation, phospholipids have been shown to enhance skin barrier and display the anti-inflammatory effect by regulating the covalently bound ω-hydroxy ceramides in the epidermis and decreasing the gene expression of both thymus activation-regulated chemokine (TARC) and thymic stromal lymphopoietin (TSLP) [42]. Even without penetrating deeper into the epidermis, the occlusive effect of the plant oil topical application decreases the loss of water from the SC and regulates keratinocyte proliferation [43].

Plant oils can be classified into essential oils and fixed oils. This article focuses only on fixed oils, which are not volatile at room temperature. Although there are different ways to obtain plant oils, cold-pressed plant oils have better nutritive properties than those that have undergone the refining process. This is because cold-pressing procedure does not involve heat or chemical treatments, which may alter their composition and therapeutic effects. Fixed plant oil components include triglycerides, FFAs, tocopherols, sterols, stanols, phospholipids, waxes, squalene, phenolic compounds [44], etc. These different compounds, when topically applied, influence skin physiology (skin barrier, inflammatory status, antioxidant response, and proliferation) differently.

Plant oils also vary by the type and the amount of triglycerides and FFAs, e.g., straight-chain saturated fatty acids (SFAs) and unsaturated fatty acids (UFAs). Topical applications of SFAs and UFAs in healthy volunteers showed differences in TEWL and irritant skin response [45]. Since composition and concentration of SFAs and UFAs are important in topical products, it is important to characterize them in each type of plant oil. Particularly, UFAs show different physiological responses when topically applied compared to TEWL [45]. Linoleic acid, for example, has a direct role in maintaining the integrity of the water permeability barrier of the skin [46,47]. The major metabolite of linoleic acid in the skin is 13-hydroxyoctadecadienoic acid (13-HODE), which possesses anti-proliferative properties [3]. In contrast, oleic acid is detrimental to skin barrier function [48]. Oleic acid causes barrier disruption and eventually induces dermatitis under continuous topical application [48]. In addition to their role in skin barrier restoration/disruption, enriched FFA plant oils have also been studied as penetration enhancers (e.g., transepidermal drug delivery). Research has suggested that oils composed mostly of monounsaturated oleic acid increased skin permeability more than oils containing an almost even mixture of both monounsaturated and polyunsaturated fatty acids. Viljoen et al. has suggested that the lipid penetration within the epidermis follows the order: olive oil > coconut oil > grape seed oil > avocado oil [49]. Moreover, the concentration of FFAs such as oleic acid with respect to triglycerides correlates with clinical measures of skin barrier function (TEWL). This ratio determines molecular interactions with SC lipids and the extent of their penetration within the epidermis [36].

Poly- and monounsaturated fatty acids may influence the inflammatory responses either as soluble lipoic mediators or in the form of phospholipids anchored in the cell membrane. Topical applications of linolenic (n-3), linoleic (n-6), and oleic (n-9) FFAs can modulate the closure of surgically induced skin wounds [50]. n-9 FFAs induced faster wound closure when compared to n-3, n-6, and control [50]. In fact, n-9 FFAs strongly inhibited the production of nitric oxide at the wound site. A mild improvement on wound closure was observed in the n-6 FFA-treated animals, correlating with a peak in nitric oxide production at 48 hours post-operatively. n-3 FFAs treatment significantly delayed wound closure, which correlates to a peak in nitric oxide at three hours post-operatively [50]. According to a previous study about the administration of pequi (*Caryocar brasielense*) almond oil in an acute hepatic injury model in rat, topical applications of poly- and monounsaturated FFAs may have a relevant role and potential therapeutic implication on WH through their modulatory effects on the inflammation rather than effects on cellular proliferation [51].

Unsaponifiables are also essential in the biological function of plant oils [44]. They have a high potential for antioxidant activity. Antioxidant activity is derived from tocopherols, carotenoids, triterpenes, flavonoids, and phenolic acids that protect from ROS [33,34,35].

### 2.1. Phenolic Compounds

Phenolic compounds are present in all vegetable oils in different concentrations. Phenolic compounds are the main antioxidants found in virgin olive oil, a well characterized oil known for its health benefits. These compounds are very important for the oxidative stability of the PUFAs within the oil. The main phenolic subclasses present in olive oil are phenolic alcohols, phenolic acids, flavonoids, lignans, and secoiridoids [52]. Another plant oil, grape seed oil, contains a large amount of similar phenolic compounds, including flavonoids, phenolic acids, tannins, and stilbenes [53]. The main polyphenols in grape seed oil are catechins, epicatechins, trans-resveratrol, and procyanidin B1 [54].

### 2.2. Triterpenes

**Figure 1:**
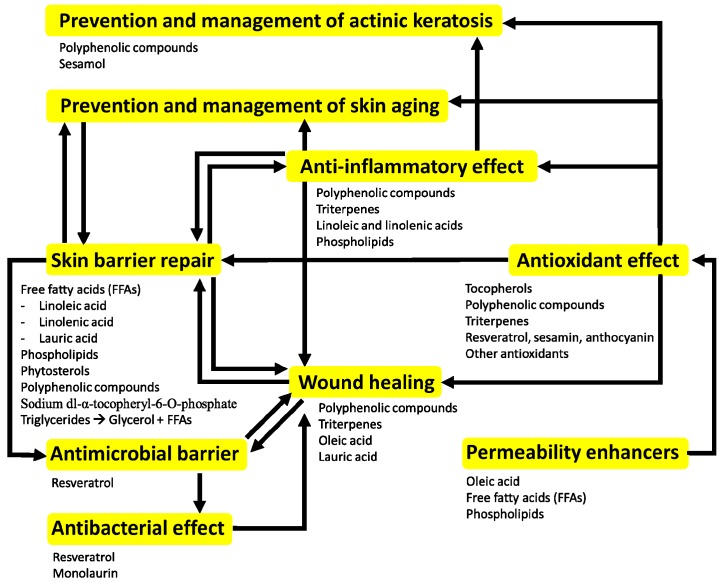
The potential benefits of plant oil topical application are diverse. Physiological responses are a result of the interaction between the bioactive compounds and the pathophysiological context of the skin.

Triterpenes have been found in many plant species, and are usually present in a small fraction of plant oil constituents. This group of compounds contains a wide range of molecules that participate in many biological reactions. Triterpenes may induce cell migration, proliferation, and collagen deposition [55]. Triterpenes also enhance the tissue repair by reducing the length of time for wound closure, and modulating the production of ROS in the wound microenvironment [55]. Betulin, a form of triterpene, is a major component of birch bark, which has been clinically shown to accelerate acute WH. Betulin treatment in keratinocyte cultures has shown to increase mRNA levels of chemokines, pro-inflammatory cytokines, and mediators that are important in WH (Figure 1). Upregulation of these crucial cytokines and mediators at the protein level were also demonstrated [56]. Of note, triterpenes are also abundant in shea butter, a popular plant oil used in skin care products and cosmetics [57].

## 3. The Potential Beneficial Effects of Topical Application of Plant Oils on Skin

The following sections review the current evidence on some plant oils and their in vivo and ex vivo effects on the skin homeostasis (Table 1).

### 3.1. Olive Oil

Olive oil comes from the fruits of *Olea europaea* trees. It consists mainly of oleic acid, with smaller quantities of other fatty acids such as linoleic acid and palmitic acid. More than 200 different chemical compounds have been detected in olive oil, including sterols, carotenoids, triterpenic alcohols, and phenolic compounds. Hydrophilic phenols are the most abundant antioxidants of olive oil. The phenolic contents have antioxidant properties higher than those of vitamin E. In fact, these phenolic compounds and their antioxidant activity exhibit anti-inflammatory properties when olive oil is included in regular diet [58]. Unsurprisingly, olive oil has been used as a skin product and hair cosmetic for a long time in several cultures. Studies on mice have shown that topical application of olive oil on pressure ulcers improves WH through the effects of anti-inflammation, reducing oxidative damage, and promoting dermal reconstruction [59]. In rat experiments, wound contraction of full-thickness burns occurred faster with olive oil treatment when compared to the silver sulfadiazine and normal saline (control) group. Studies have also shown that concomitant use of other oils such as buckthorn oil with olive oil have positive effects on the skin [114]. In a randomized controlled trial by Zahmatkesh et al., a mixture of olive oil, sesame oil, and honey was demonstrated to be a useful treatment for burns, by preventing infections, accelerating tissue repair, and facilitating debridement [60]. Moreover, in a murine study with UVB radiation, Ichihashi et al. found that extra virgin olive oil applied to the skin delayed the onset and reduced the incidence of skin cancer development, likely secondary to reduced number of 8-hydroxy-2′-deoxyguanosine (8-OHdG) positive cell formation (a biomarker of oxidative stress and carcinogenesis) [61]. It has also been demonstrated that daily consumption of olive oil phenolics protect from DNA oxidation in postmenopausal women [115] and interfere with G1 cell cycle in human colon adenocarcinoma cells and promyelocytic leukemia cells [116].

In contrast to its positive role in WH promotion and reducing skin cancer development, topically applied olive oil has a detrimental effect on SC integrity and skin barrier function [62,117]. There is evidence of increased TEWL after topical application to the skin of the forearms of adult volunteers with and without AD [62]. Experiments on mice also elicited similar results [117]. Although skin barrier restoration is a key event in WH, olive oil may promote WH by modulating early phases such as inflammation, and stimulating dermal reconstruction, both of which are not related to subsequent re-epithelialization and the consequent permeability barrier restoration. At the present, it is widely accepted that minor components of olive oil also exert potent anti-inflammatory activities [58].

Olive pomace oil, a natural by-product of olive oil production, has also been found to contain minor constituents with antioxidant, antithrombotic, and antiatherogenic activities when it is included in the regular diet [58]. However, the effects of olive pomace oil in the skin have not been characterized yet.

### 3.2. Sunflower Seed Oil

Sunflower seed oil originates from the seeds of *Helianthus annus*. Sunflower oil has been recommended for the removal of hot tar in patients of tar burn [118]. The components of sunflower oil mainly consist of oleic and linoleic acids. Sunflower seed oil contains relatively higher linoleic acid concentration relative to olive oil. This property makes sunflower oil a suitable ingredient in skin products due to the positive benefits of linoleic acid [117]. Sunflower seed oil has been shown to preserve SC integrity and improve hydration of the adult skin without inducing erythema [62]. Linoleic acid serves as an agonist at peroxisome proliferator-activated receptor-alpha (PPAR-α), which enhances keratinocyte proliferation and lipid synthesis [62]. This in turn enhances skin barrier repair. Natural oils such as sunflower, sesame, or safflower seed oil have been suggested as good options for their use in promoting skin barrier homeostasis [119]. However, in a pilot study conducted by Cooke et al. of neonatal skin topically treated with sunflower seed oil or olive oil, there were no differences in lipid structure changes, TEWL, hydration, skin surface pH, erythema, or skin assessment scores between the olive oil and sunflower oil groups [63]. According to Norlen (2016), however, this well-conducted pilot study needs a long-term observational study to investigate whether the topical application of oils from birth may contribute to the development of atopic eczema [64]. Moreover, in contrast to delayed improvement in lipid ordering observed after topical application of olive oil, this feature was not observed after application of sunflower seed oil [64]. Sunflower seed oil also exhibited a chemopreventive effect in a murine model of skin cancer with two-stage carcinogenesis. Sesamol, one of its constituent, specifically play a role in the chemopreventive effects [66].

### 3.3. Grape Seed Oil

Grape seed oil comes from the seeds of *Vitis vinifera*. It is rich in phenolic compounds, FFAs, and vitamins. Grape seed oil has been evaluated for WH activity in rat models. Shivananda Nayak et al. has shown that the hydroxyproline content of the granulation tissue was significantly higher in the animals treated with the grape oil [67]. Additionally, the rate of wound closure was also quicker, suggesting their WH potential [67]. However, the direct topical application of grape seed oil on human skin has not yet been well investigated. Grape seed proanthocyanidin extract, which contains resveratrol, has been topically applied to mice, showing faster wound contraction, enhanced synthesis of vascular endothelial growth factor (VEGF), and greater connective tissue deposition [68]. Resveratrol displays a direct antimicrobial activity against pathogens, such as *Staphylococcal aureus*, *Enterococcus faecalis*, and *Psedomonas aeruginosa* [69]. Topically applied resveratrol increases cathelicidin production in normal skin [70]. Cathelicidin is one of the inducible antimicrobial peptides and inhibits the growth of *Staphylococcal aureus* [70]. Studies are currently being done to successfully encapsulate and preserve resveratrol from grape seed oil [71]. In addition to resveratrol, grape seed oil has a high content of linoleic acid, vitamin E, and phenolic compounds. Phenolic compounds, resveratrol, and vitamin E in grape seed oil provide most of its antioxidant activity. Moreover, phytosterols present in grape seed oil may modulate pro-inflammatory mediators [72].

### 3.4. Coconut Oil

Coconut oil is extracted from the kernel or meat of mature coconuts harvested from the coconut palm (*Cocos nucifera*). Coconut is composed of many FFAs including lauric acid (49%), myristic acid (18%), palmitic acid (8%), caprylic acid (8%), capric acid (7%), oleic acid (6%), linoleic acid (2%), and stearic acid (2%) [6]. Coconut oil has been shown to be as effective and safe as mineral oil when applied as moisturizers for mild to moderate xerosis [120]. In a study of pediatric patients with mild to moderate AD, topical applications of virgin coconut oil was shown to be effective in decreasing the severity of the disease, ameliorating disease severity index (SCORAD), and improving barrier function (TEWL and skin capacitance) [73]. Topical applications of virgin coconut oil are effective in promoting WH through faster epithelization. A histopathological study by Nevin et al. revealed increased neovascularization, fibroblast proliferation, pepsin-soluble collagen synthesis, and turnover of collagen in wounds [74]. Kim et al. demonstrated that coconut oil increased expression of CE components, thereby contributing to protective barrier functions of the SC [75]. Furthermore, the expression of inflammatory profile was lower in the coconut oil-treated group after exposure to UVB radiation [75]. Topical coconut oil protects the skin from UV radiation [65].

Of all the acid components of coconut oil, monolaurin has been shown to have additional significance. Monolaurin is a monoglyceride derived from lauric acid. It comprises nearly 50% of coconut’s fat content. Monolaurin displays antimicrobial activity by disintegrating the lipid membrane of lipid-coated bacteria including *Propionibacterium acnes*, *Staphylococcus aureus*, and *Staphylococcus epidermidis* [76]. Coconut oil in concentrations of 5% to 40% (*w*/*w*) exhibited bactericidal activity against *Pseudomonas aeruginosa, Escherichia coli, Proteus vulgaris*, and *Bacillus subtilis* [77]. Cellular studies have also shown that monolaurin exhibits antiviral and antifungal activity [78].

### 3.5. Safflower Seed Oil

Safflower seed oil comes from the seeds of the *Carthamus tinctorius*. It contains a large amount of the polyunsaturated linoleic acid (70%) and monounsaturated oleic acid (10%), and lesser amounts of stearic acid. Safflower has been shown to be a very good analgesic and antipyretic. Modern pharmacological studies demonstrated that the extracts of safflower had several physiological functions, such as anticoagulation, vasodilation, antioxidation, melanin production inhibition, immunosuppression, and antitumor activity. For example, the flavone luteolin and its glucopyranoside have been reported to exert anti-inflammatory effects at concentrations in the low micromolar range [79,80,121]. This anti-inflammatory effect is explained by inhibition of NF-κB activity [80]. Fatty acid constituents of topical applied plant oils may modify the fatty acid profiles of the babies. Solanki et al. have showed that topically applied safflower seen oil is readily absorbed in neonates and probably it has nutritional benefits [122]. Fatty acid profiles showed significant rise in linolenic acid and arachidonic acid under topical safflower oil treatment [122]. Since the metabolism of PUFAs by skin epidermal enzymes is related to the generation of anti-inflammatory molecules, the modification in fatty acid profiles might be of interest in clinical practice [3].

### 3.6. Argan Oil

Argan oil is produced from the kernels of *Argania spinosa* L. Argan oil is composed of mono-unsaturated (80%) and saturated (20%) fatty acids. It contains polyphenols, tocopherols, sterols, squalene, and triterpene alcohols. Traditionally, argan oil has been utilized in cooking, in the treatment of skin infections, and in skin/hair care products. Daily topical application of argan oil has also been shown to improve skin elasticity [81] and skin hydration by restoring the barrier function and maintaining the water-holding capacity [123]. Additionally, topical applications onto skin provide a softening and relaxing effect on the skin, as well as helping to facilitate the accumulation and transdermal delivery of topical drugs such as allantoin [82]. Recently, tocopherol-rich argan oil-based nanoemulsions has been developed as vehicles possessing anticancer activity in murine breast and colon carcinoma cells [83]. Argan oil has also been shown to be effective in enhancing WH created second-degree burns in rats [84].

### 3.7. Soybean Oil

Soybean oil is a vegetable oil extracted from the seeds of the *Glycine max*. Most research on soybean oil have been conducted on its extracts. Topical application of soybean oil extracts has been shown to decrease the TEWL of forearm skin [11]. This feature may be linked to the presence of soy phytosterols, which have shown a positive effect on skin barrier recovery [124]. On the other hand, anthocyanin contents in the seed coat of black soybean were shown to have anti-human tyrosinase activity and antioxidative activity [85]. Black soybean anthocyanins attenuate inflammatory responses by suppressing ROS production as well as mitogen activated protein kinases that are important in the signaling of lipopolysaccharide-stimulated macrophages [86]. Moreover, topical soybean oil protects against UVB-induced cutaneous erythema [87].

### 3.8. Peanut Oil

Peanut oil has been shown to have hydrating effects in human skin without significantly increasing TEWL [88]. Topical peanut oil protects the skin from UV radiation [65]. Lasne et al. also showed the inhibition of chemically-induced skin carcinogenesis in mice treated with topical peanut oil [89].The increasing prevalence of peanut allergy has led to new discussion about the safety of topical preparations containing peanut oil. However, research has suggested that the refined peanut oil-containing preparation is safe for topical use, even in persons who are sensitive to peanuts [125,126].

### 3.9. Sesame Oil

Sesame oil is derived from *Sesamum indicum*. Sesame oil has been incorporated in many food items in the past 6000 years. Sesame seeds contain significant amounts of lignans such as sesamin, sesamolin, and sesaminol [127], all of which exhibit antioxidative activity. Sesamin is highly hydrophobic. A significant positive correlation was observed between the oil content of sesame seed and the sesamin content in the oil [128]. Research has shown that the topical use of sesame oil might attenuate oxidative stress by inhibiting the production of xanthine oxidase and nitric oxide in rats [90]. Sesame oil has been used in traditional Taiwanese medicine to relieve the inflammatory pain of joints and wounds. Massage with topical sesame oil has shown to be effective in significantly reducing pain severity of patients with limb trauma [91]. In a rat model of monosodium urate monohydrate (MSU) crystal-induced acute inflammatory response in a pseudosynovial cavity, orally administered sesame oil reduced inflammation [92]. In a clinical study by Shamloo et al., topical application of sesame oil was shown to lower the severity of pain and reduce the frequency of nonsteroidal anti-inflammatory drug use in patients with limb trauma [93]. Topical sesame oil also protects the skin from UV radiation [65]. In addition, sesame oil showed a chemopreventive effect in a murine model of skin cancer with two-stage carcinogenesis. Its constituent, sesamol, has also been demonstrated to play a role in chemoprevention [66].

### 3.10. Avocado Oil

Avocado oil is derived from the fruit of the *Persea americana*. Avocado oil extracted from the pulp of the fruit is rich in linoleic acid (6.1–22.9%), linolenic acid (0.4–4.0%), and oleic acid (31.8–69.6%). It also contains β-sitosterol, β-carotene, lecithin, minerals, and vitamins A, C, D, and E [94]. It is an excellent source of enrichment for dry, damaged, or chapped skin [11]. Research has been conducted on the effect of topical administration of avocado fruit extract on wound models in rats, revealing faster re-epitheliazation and higher hydroxyproline content of the repaired wound [95]. The topical application of avocado oil in rats has also been shown to increase collagen synthesis and decrease the numbers of inflammatory cells during the WH process [94,96].

### 3.11. Borage Oil

Borage oil is derived from the seeds of the *Borago officinalis*. Borage oil contains high levels of the ω-6 series essential fatty acids that are important in skin structure and function [129]. The linoleic acid in borage oil contributes to its therapeutic actions in AD. Topical application of borage oil in infants and children with seborrheic dermatitis or AD has been shown to normalize skin barrier function [130]. A double-blind, placebo-controlled clinical trial was performed to test clinical effects of undershirts coated with borage oil on children with AD [97]. In the group treated with borage oil, TEWL on the skin of back decreased. Additionally, no side effects were found in these subjects [97].

### 3.12. Jojoba Oil

Jojoba (*Simmondsia chinensis*) is a long-lived, drought resistant, perennial plant. Jojoba oil exhibits a high oxidative stability and resistance to degradation [131]. Jojoba oil is widely used in cosmetic formulas such as sunscreens and moisturizers. It has been shown to be effective in enhancing the absorption of topical drugs [132,133,134]. The high content of wax esters makes jojoba oil a good repair option for dermatoses with altered skin barriers, such as seborrheic dermatitis, eczematous dermatitis, AD, and acne [98]. Jojoba oil also has a proven anti-inflammatory effect, with potential uses in a variety of skin conditions including skin infections, skin aging, and WH [99,132].

### 3.13. Oat Oil

Oat oil originates from *Avena sativa*. It consists of 36–46% linoleic and 28–40% oleic acid [135]. Oat in colloidal form is a centuries-old topical treatment for a variety of skin conditions, including skin rashes, erythema, burns, itch, and eczema. Although oleic acid may disrupt skin barrier [62,117], the high percentage (36–46%) of linoleic acid may contribute to the final effect of oat oil on barrier repair [47,100]. Colloidal oat extracts exhibit direct anti-oxidant and anti-inflammatory activities, which may explain the efficacy of lotions containing colloidal oatmeal [101]. Avenanthramides are phenolic compounds present in oats. Avenanthramides inhibit activation of NF-κB and reduce inflammation by inhibiting cytokines [102]. In vitro studies have shown that oat oil can upregulate the expression of differentiation genes (e.g., involucrin, small prolin-rich protein family (SPRRs), and transglutaminase 1) and ceramide processing genes (β-glucocerebrosidase, sphingomyelinases 3 and ABCA12) in keratinocytes [103]. In addition, oat oil treatment in keratinocytes was shown to have significantly increased ceramide levels (70%) through the activation of peroxisome proliferator-activated receptors (PPARs) [103].

### 3.14. Pomegranate Seed Oil

Pomegranate seed oil comes from the seed of *Punica granatum*. It is a good source of essential FFAs, phenolic compounds, phytosterols, and lipid-soluble fractions [136]. Pomegranate seed oil contains 63% UFA, including linoleic acid (29%) and oleic acid (10%) [136]. Pomegranate seed oil is well known for its high concentration of polyphenolic compounds and for its antioxidant and anti-inflammatory properties. An oil-in-water cream containing pomegranate seed oil and *C. lechleri* resin extract can be helpful in the prevention or improvement of skin changes associated with striae [137]. Pomegranate seed oil has been used in nanoemulsions to facilitate the delivery of pomegranate peel polyphenols [138]. Nanoemulsions with pomegranate seed oil has been shown to improve both photostability and in vivo anti-nociceptive effect of ketoprofen [139]. A study of CD_1_ mice with topically applied pomegranate seed oil has shown that pomegranate seed oil (5%) significantly decreased tumor incidence and 12-*O*-tetradecanoyl-phorbol-13-acetate (TPA)-induced ornithine decarboxylase activity in the chemical-induced skin cancer model. The results highlighted the potential of pomegranate seed oil as a chemopreventive agent against skin cancer [104].

### 3.15. Almond Oil

Almond oil comes from *Oleum amygdalae*. Almond oil has emollient and sclerosant properties, which have been used to improve complexion and skin tone. In a nonrandomized study, Tashan and Kafkasli (2012) have demonstrated that massage with bitter almond oil may be effective in reducing the visibility of current striae gravidarum, and in the prevention of new striae [105]. Other formulations have been shown to ameliorate striae itching [106]. However, other products containing almond oil have not shown to have similar benefit [140]. For example, sweet almond oil in creams are more effective than the base cream at ameliorating the itching of striae and preventing its progression [106]. In a study by Sultana et al. done with murine models, topical almond oil was shown to prevent the structural damage caused by UV irradiation [107].

### 3.16. Bitter Apricot Oil

In Eastern medicine, bitter apricot seed (*Semen Armeniacae amarum*) has been traditionally used to treat skin diseases. Bitter apricot oil has been shown to induce apoptosis of HaCaT cells through both death receptor and mitochondrial pathways. Apoptosis has been shown to correlate with inhibition of the NF-κB pathway [108]. It has been suggested that apricot oil may be a potential candidate for psoriasis treatment given its pro-apoptotic effect on human keratinocytes [108].

### 3.17. Rose Hip Oil

Rose hip oil is extracted from seeds of rose hip (*Rosa canina* L.). Rose hip oil contains substantial UFAs. The most abundant fatty acid is linoleic acid (35.9–54.8%), followed by α-linolenic acid (16.6–26.5%), and oleic acid (14.7–22.1%) [141]. An appreciable number of lipophilic antioxidants is present, especially the tocopherols and carotenoids. Rose hip oil also contains high level of phenolic acids, especially p-coumaric acid methyl ester, vanillin, and vanillic acid. Due its high composition of UFAs and antioxidants, this oil has relatively high protection against inflammation and oxidative stress [109]. Shabikin et al. has tested the efficacy of topical rose hip seed oil together with an oral fat-soluble vitamins on different inflammatory dermatitis such as eczema, neurodermatitis, and cheilitis, with promising findings of the topical use of rose hip seed oil on these inflammatory dermatoses [110].

### 3.18. German Chamomile Oil

German chamomile oil comes from *Matricaria recutita*. In a study with the murine AD model, serum IgG1 and IgE levels were significantly decreased in the group treated with German chamomile oil application. Topical application of this oil was associated with lower serum histamine level and decreased frequency of scratching among subjects. The result demonstrated the immune-regulatory potential of German chamomile oil for alleviating AD through modulation of Th2 cell activation [111].

### 3.19. Shea Butter

Shea butter is extracted from the kernels of the sheu tree (*Vitellaria paradoxa*). Shea butter is composed of triglycerides with oleic, stearic, linoleic, and palmitic fatty acids, as well as unsaponifiable compounds [142]. Shea butter is frequently used in the cosmetic industry due to its high percentage of the unsaponifiable fraction (e.g., triterpenes, tocopherol, phenols, and sterols), which possesses potent anti-inflammatory and antioxidant properties [57]. In the study of lipopolysaccharide-activated macrophage cells, shea butter exhibited anti-inflammatory effects through inhibition of iNOS, COX-2, and cytokines via the NF-κB pathway [112]. Additional research on AD has shown that the cream containing shea butter extract had the same efficacy as ceramide-precursor product [113].

## 4. Conclusions

Topical applications of plant oils may have different effect on the skin according to their composition and the pathophysiological context of the skin. The composition varies by different extraction methods. When applied topically, constituents of plant oils (triglycerides, phospholipids, FFAs, phenolic compounds and antioxidants) may act synergistically by several mechanisms: (i) promoting skin barrier homeostasis; (ii) antioxidative activities; (iii) anti-inflammatory properties; (iv) direct and indirect (upregulation of antimicrobial peptides) anti-microbial properties; (v) promoting wound healing; and (vi) anti-carcinogenic properties. Future studies can add to current findings to allow for better understanding of these oils, with the potential to develop dermatological treatments and skin care products using these oils.

## Figures and Tables

**Table 1:** The effects of topically applied plant oils on skin pathology.

Plant Oils	Skin Barrier Repair	Anti-Bacterial Effect	Anti-Inflammatory Effect	Antioxidant Effect	Wound Healing	Skin Aging	Skin Cancer	References
Olive oil	No	?	Yes	Yes	Yes	Possible effect	Yes	Cardoso CR et al., 2004 [50] Nasopoulou C et al., 2014 [58] Donato-Trancoso A et al., 2016 [59] Zahmatkesh M et al., 2015 [60] Budiyanto A et al., 2000 [61] Danby SG et al., 2013 [62] Cooke A et al., 2016 [63] Norlen L et al., 2016 [64] Korac RR et al., 2011 [65]
Sunflower seed oil	Yes	?	Yes	?	Possible effect	?	Yes	Cardoso et al., 2004 [50] Danby SG et al., 2013 [62] Cooke A et al., 2016 [63] Norlen L et al., 2016 [64] Kapadia GJ et al., 2002 [66]
Grape seed oil	?	Yes	Possible effect	Yes	Yes	Possible effect	Possible effect	Kapadia GJ et al., 2002 [66] Shivananda Nayak B et al., 2011 [67] Khanna S et al., 2002 [68] Chan MM et al., 2002 [69] Park K et al., 2013 [70] Davidov-Pardo G et al., 2015 [71] Shinagawa FB et al., 2015 [72]
Coconut oil	Yes	Yes	Yes	Yes	Yes	Yes	?	Evangelista MT et al., 2014 [73] Nevin KG et al., 2010 [74] Kim S et al., 2017 [75] Korac RR et al., 2011 [65] Preuss HG et al., 2005 [76] Oyi AR et al., 2010 [77] Esquenazi D et al., 2002 [78]
Safflower seed oil	?	?	Yes	?	?	?	?	Chen CY et al., 2007 [79] Lopez-Lazaro M, 2009 [80]
Argan oil	Yes	?	Yes	?	Yes	?	Possible effect	Boucetta KQ et al., 2015 [81] Manca ML et al., 2016 [82] Jordan M et al., 2012 [83] Avsar U et al., 2016 [84]
Soybean oil	Yes	Yes	Yes	Yes	?	?	?	Patzelt A et al., 2012 [11] Jhan JK et al., 2016 [85] Kim JN et al., 2017 [86] Bonina F et al., 2005 [87]
Peanut oil	Yes	?	?	?	?	Yes	Yes	Korac RR et al., 2011 [65] Zhai H et al., 2003 [88] Lasne C et al., 1991 [89]
Sesame oil	Possible effect	?	Yes	Yes	?	Yes	Yes	Kapadia GJ et al., 2002 [66] Korac RR et al., 2011 [65] Chiang JP et al., 2005 [90] Nasiri M et al., 2017 [91] Hsu DZ et al., 2013 [92] Bigdeli Shamloo et al., 2015 [93]
Avocado oil	?	?	Possible effect	?	Yes	?	?	Patzelt A et al., 2012 [11] De Oliveira AP et al., 2013 [94] Nayak BS et al., 2008 [95] Lamaud E et al., 1982 [96]
Borage oil	Yes	?	Possible effect	?	?	?	?	Kanehara S et al., 2007 [97]
Jojoba oil	Yes	Possible effect	Yes	Yes	Yes	Yes	?	Meier L et al., 2012 [98] Ranzato E et al., 2011 [99]
Oat oil	Yes	Possible effect	Yes	Yes	?	?	?	Nebus J et al., 2009 [100] Reynertson KA et al., 2015 [101] Sur R et al., 2008 [102] Chon SH et al., 2015 [103]
Pomegranate seed oil	?	?	?	Yes	?	Possible effect	Possible effect	Hora J et al., 2003 [104]
Almond oil	Possible effect	?	?	?	?	Yes	?	Timur Tashan S et al., 2012 [105] Hajhashemi M et al., 2017 [106] Sultana Y et al., 2007 [107]
Bitter apricot oil	?	?	?	?	?	?	Possible effect	Li K et al., 2016 [108]
Rose hip oil	Possible effect	?	Yes	Yes	?	Yes	?	Chrubasik C et al., 2008 [109] Shabykin GP et al., 1967 [110]
German chamomile oil	Possible effect	?	Yes	?	?	?	?	Lee SH et al., 2010 [111]
Shea butter	Possible effect	?	Yes	Yes	?	?	?	Verma N et al., 2012 [112] Hon KL et al., 2015 [113]

When there is no concrete evidence for the specific effect of topical treatment of some plant oils, it is indicated with “?”.

## Abbreviations

15-HETE AD	15-hydroxyeicosatetraenoic acid Atopic dermatitis
CE	Cornified envelope
COX	Cyclooxygenase
FFA	Free Fatty Acid
LB	Lamellar body
MMP	Matrix metalloproteinase
NMF	Natural moisturizing factor
PMN	Polymorphonuclear neutrophils
PPAR PUFA	Peroxisome proliferator-activated receptor Polyunsaturated fatty acid
ROS	Reactive oxygen species
SC	Stratum corneum
SFA	Saturated Fatty Acid
SG	Stratum granulosum
TEWL	Transepidermal water loss
TNF-α	Tumor necrosis factor-alpha
UFA	Unsaturated Fatty Acid
UV	Ultraviolet
WH	Wound healing
